# Intestinal inflammation and stem cell homeostasis in aging *Drosophila melanogaster*

**DOI:** 10.3389/fcimb.2013.00098

**Published:** 2013-12-16

**Authors:** Arshad Ayyaz, Heinrich Jasper

**Affiliations:** Buck Institute for Research on AgingNovato, CA, USA

**Keywords:** stem cell, tissue homeostasis, aging, dysbiosis, dysplasia

## Abstract

As a barrier epithelium, the intestinal epithelium has to coordinate physiological functions like digestion and nutrient resorption with the control of commensal bacteria and the prevention of pathogenic infections. It can therefore mount powerful innate immune and inflammatory responses, while, at the same time, maintaining tissue homeostasis through regenerative processes. How these different functions are coordinated remains unclear, and further insight is required to understand the age-related loss of homeostasis in this system, as well as the etiology of inflammatory and proliferative diseases of the gut. Recent work in *Drosophila melanogaster* has provided important new insight into the regulation of regenerative activity, innate immune homeostasis, commensal control, as well as age-related dysfunction in the intestine. Interestingly, many of the identified processes and mechanisms mirror similar homeostatic processes in the vertebrate intestine. This review summarized the current understanding of how innate immune responses, changes in commensal bacteria, and other challenges influence regenerative activity in the aging intestinal epithelium of flies and draws parallels to similar processes in mammals.

## Introduction

As a major barrier epithelium, the intestinal epithelium is the first line of defense against pathogenic microorganisms, while at the same time managing the beneficial interaction between commensal bacteria and the host. Accordingly, it mounts highly coordinated and regulated stress and immune responses to govern these interactions. Dysfunction in these signaling mechanisms can cause intestinal dysbiosis and chronic inflammation, and these pathologies can in turn negatively influence epithelial homeostasis, causing dysplasias and cancers (Gonda et al., 2009; Uronis et al., 2009; Kaser et al., 2010; Niwa et al., 2010; Clemente et al., 2012; Kostic et al., 2012). Deeper insight into the interaction between the intestinal epithelium, the commensal microbiota, and stress and innate immune signaling in epithelial cells is thus paramount to developing rational therapies and preventive strategies for these diseases. Such insight is further expected to significantly contribute to our understanding of changes in tissue homeostasis in the aging organism.

Elderly individuals are more susceptible to infectious diseases, including inflammatory disorders (Clemente et al., 2012), colorectal cancer (Patel et al., 2009), metabolic imbalance (Roberts and Rosenberg, 2006) and gastrointestinal infections (Duncan and Flint, 2013). Interestingly, various other age-related physiological complications, for instance obesity (Kallus and Brandt, 2012), insulin resistance (De Bandt et al., 2011), and general frailty (Claesson et al., 2012) have been associated with changes in the intestinal microbiota, suggesting that age-related changes in epithelial/commensal interactions impact not only inflammatory diseases of the gut, but potentially overall health and lifespan.

Recent advances in sequencing techniques that allow “metagenomic” strategies have revolutionized the study of microbiota associated with the human intestine (Qin et al., 2010; Kamada et al., 2013; Koeth et al., 2013; Stecher et al., 2013). An average human gut harbors as many as 10^14^ bacterial cells belonging to 400–1000 different species. Composition of this microbiota is highly variable among individuals and changes along the lifespan of individuals (Biagi et al., 2010; Claesson et al., 2011, 2012; Lozupone et al., 2012; Schloissnig et al., 2013). At the same time, the composition of the microbiota is remarkably stable in the short term (Power et al., 2013), suggesting that a tightly controlled immune response maintains a diverse array of “commensals” while simultaneously eliminating hazardous microbes in healthy intestines.

Age-related changes in microbiota composition are thus likely a consequence of changes in the ability of the intestinal epithelium to properly control the type and number of microorganisms colonizing the gut. These changes are in turn expected to be caused by deregulation of epithelial signaling events and by a breakdown of epithelial homeostasis that occur due to common age-associated cellular changes. Broadly, the aging process is characterized by the loss of proteostasis, accumulation of DNA damage, increased oxidative stress, metabolic imbalances and deregulated stress signaling (Paaby and Schmidt, 2008; Karpac and Jasper, 2009; Kenyon, 2010). While the progression toward age-related dysfunction in the intestinal epithelium remains unclear, it can be anticipated that the damage to epithelial cells resulting from such general age-associated molecular changes is likely to affect epithelial interactions with commensal microbial communities. At the same time, these changes also cause increased vulnerability to pathobionts in older guts (Biagi et al., 2012; Schloissnig et al., 2013). The resulting chronic stimulation of immune and inflammatory responses is further likely to promote tissue dysfunction by impacting regenerative and homeostatic processes.

The interactions between microbiota, stress and immune signaling in epithelial cells, as well as regenerative processes in the epithelium, represent a complex and wide-ranging field of study, and simple animal models are needed to provide fundamental insight into these interactions. The availability of powerful genetic tools for *D. melanogaster*, as well as its short lifespan and the relative simplicity of its intestine, but also the presence of complex epithelial interactions with commensals and of regenerative processes that resemble similar processes in mammals, have recently elevated the fly to a model organism of choice in this context. A large number of studies have already provided important mechanistic insight into epithelial/commensal interactions, as well as age-related changes in these interactions and their consequences for epithelial homeostasis (Buchon et al., 2009a, 2013a; Apidianakis and Rahme, 2011; Hochmuth et al., 2011; Karpac et al., 2011; Rera et al., 2011; Lee and Brey, 2013).

In the wild, *D. melanogaster* feeds on rotten fruits and vegetables. Such feeding behavior exposes them to repeated interactions with a variety of microbes. Distinct mechanisms have therefore evolved in fruit flies that enable them to maintain intestinal tissue homeostasis and survive in a microbe-rich environment (Ferrandon, 2013). In older flies, however, a widespread growth of intestinal microbial populations is associated with hyperplasia and misdifferentiation of intestinal stem cells (ISCs) and their progeny, leading to loss of tissue homeostasis (Buchon et al., 2009a; Biteau et al., 2010). Interestingly, over-expression of stress-protective genes in ISCs is sufficient to rescue this age-related loss of homeostasis and to increase *Drosophila* lifespan (Biteau et al., 2010; Rera et al., 2011, 2012). This finding supports the notion that managing the loss of intestinal homeostasis is critical for health and lifespan in metazoans, and highlights the usefulness of flies as models for inflammatory diseases of the gut. In this review we will summarize the current understanding of the interaction between innate immune responses, commensal microbiota, and proliferative homeostasis in the aging intestinal epithelium in *D. melanogaster*.

## The *drosophila* intestine

**Figure 1:**
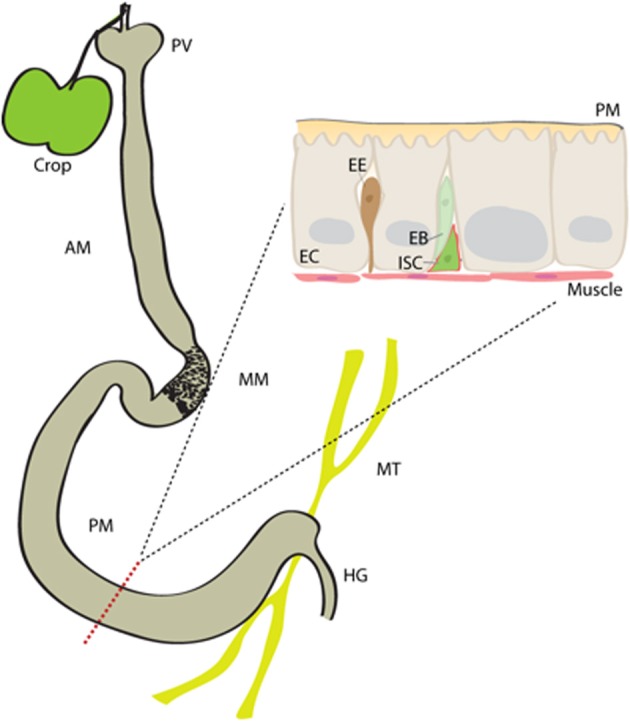
**The *Drosophila intestine***. The midgut in *Drosophila* is subdivided into anterior midgut (AM), middle midgut (MM) and posterior midgut (PM) regions. It contains single population of mitotically active intestinal stem cells (ISCs), which spread throughout from anterior to posterior regions. An ISC asymmetrically divides to generate an intermediate enteroblast (EB), which eventually differentiates either into an enterocyte (EC) or enteroendocrine (EE) cell. proventriculous (PV), hindgut (HG), Malpighian tubules (MT), peritrophic matrix (PM).

The gastrointestinal tract in *Drosophila* can be subdivided into the crop, foregut, midgut and hindgut (Figure 1). The crop is a food storage organ which is attached to the distal end of the foregut, via a thin tube. The midgut can further be divided into anterior, middle and posterior regions. The anterior midgut (AM) encompasses the proventriculus, and opens into the acidic middle midgut (MM; also called copper cell region). The posterior midgut, in turn, extends from the MM to a fusion point where it is connected to the hindgut and to malpighian tubules (Buchon et al., 2013b; Marianes and Spradling, 2013).

The *Drosophila* intestinal epithelium is a monolayer composed of three types of cells; the polyploid enterocytes (EC) form the majority of the midgut cell population, followed by hormone secreting enteroendocrine (EE) cells and the proliferating ISCs. ECs are absorptive cells but also secrete digestive enzymes in some parts of the gut, and play a central role in mounting innate immune responses to infection and in managing the commensal population. Proteases, lipases (such as LipA), carbohydratases, catalytic peptidoglycan recognition proteins (PGRPs) and lysozymes are among the digestive enzymes secreted by midgut cells (Sieber and Thummel, 2012; Lemaitre and Miguel-Aliaga, 2013). The MM, in turn, contains acid secreting copper cells, most likely to aid digestion.

Regenerative processes in the intestinal epithelium differ along the gastrointestinal tract, and are influenced by local signals in each compartment (Buchon et al., 2013b; Guo et al., in press; Li et al., 2013; Marianes and Spradling, 2013). Interestingly, this compartmentalization seems to decline in the aging intestine, causing widespread deregulation of stem cell activity (Buchon et al., 2013b).

Regeneration of the posterior midgut epithelium is best understood so far. ISCs in this area can mount rapid and widespread regenerative responses to damage. During this renewal, ISCs divide asymmetrically to produce a population of non-differentiated progenitors calles enteroblasts (EBs) (Micchelli and Perrimon, 2006; Ohlstein and Spradling, 2006). EBs are not mitotically active, and differentiate into either an EC or an EE cell, depending on differential Notch signaling activity (Ohlstein and Spradling, 2007; Biteau et al., 2011a; Perdigoto et al., 2011; Cordero and Sansom, 2012; Kapuria et al., 2012). ISCs are also known to divide symmetrically to expand their own population (O'brien et al., 2011; Goulas et al., 2012). The ISCs are located close to the basal membrane (BM) of the epithelium and are in close proximity to the surrounding circular visceral muscle. The BM and visceral muscle, but also EBs and ECs, influence ISC proliferative activity and maintenance (Bardin et al., 2010; Biteau and Jasper, 2011; Xu et al., 2011; Cordero et al., 2012a; Goulas et al., 2012; Zhou et al., 2013).

## Control of ISC proliferation

The proliferative activity of ISCs is very plastic. While low levels of homeostatic proliferation are generally observed in young, healthy guts, strong regenerative activity is observed in response to insults that damage the epithelium. EGF, Insulin/IGF (IIS), and p38MAPK signaling pathways are essential for ISC proliferation (Park et al., 2009; Biteau et al., 2010, 2011a; Biteau and Jasper, 2011). Constitutive activation of EGF receptor (EGFR) or insulin receptor (InR) increases the rate of ISC proliferation, indicating that RTK signaling can modulate ISC activity in accordance with the metabolic status of the animal (Biteau and Jasper, 2011; Karpac et al., 2011; O'brien et al., 2011; Xu et al., 2011). Long-term stem cell maintenance is further ensured by mechanisms that prevent activation of Target of Rapamycin (TOR) signaling (Amcheslavsky et al., 2011; Kapuria et al., 2012; Quan et al., 2013), and by muscle—derived Wingless (Sackton et al., 2007; Lin et al., 2008; Takashima et al., 2008; Cordero and Sansom, 2012; Cordero et al., 2012b).

While the signaling pathways listed above are required for homeostatic proliferation and maintenance of ISCs, various stress signaling pathways have been identified that govern induction of ISC proliferation when the intestinal epithelium is exposed to a stress or is injured. Stressors that trigger ISC proliferation include oxidative stress (Biteau et al., 2008; Choi et al., 2008; Buchon et al., 2009a), bacterial infection (Apidianakis et al., 2009; Buchon et al., 2009b; Cronin et al., 2009; Jiang et al., 2009; Guo et al., 2013), DNA damage (Amcheslavsky et al., 2011; Guo et al., 2013), aging (Biteau et al., 2008, 2010, 2011b; Choi et al., 2008; Buchon et al., 2009a; Karpac et al., 2009; Hochmuth et al., 2011), and factors that cause apoptosis and damage to ECs (Jiang et al., 2009; Amcheslavsky et al., 2011). Jun-N-terminal Kinase (JNK) (Biteau et al., 2008; Choi et al., 2008), JAK/Stat signaling (Buchon et al., 2009a; Cronin et al., 2009; Jiang et al., 2009) and the Hippo/Yorkie pathway (Karpowicz et al., 2010; Ren et al., 2010; Shaw et al., 2010; Staley and Irvine, 2010) are all critical for stress-induced ISC proliferation [reviewed in Biteau et al. (2011a)]. The integration of these inductive signals with signaling pathways that play a permissive role for proliferation, as well as the exact cellular interactions during a regenerative response, are only beginning to be understood. Following bacterial infection or an injury, interleukin-6-like cytokines of the Unpaired (Upd) family, especially Upd 2 and 3 are induced in and secreted by damaged and dying ECs (Jiang et al., 2009; Osman et al., 2012; Zhou et al., 2013). Upds activate JAK/Stat signaling, either in ISCs directly (Buchon et al., 2009a; Cronin et al., 2009; Jiang et al., 2009), or in visceral muscle, where it induces the EGF-like ligand Vein, which in turn stimulates ISC proliferation (Jiang and Edgar, 2009; Buchon et al., 2010; Biteau and Jasper, 2011; Xu et al., 2011; Zhou et al., 2013).

The JNK pathway also plays a dual role in stimulating ISC proliferation: JNK is activated by reactive oxygen species (ROS) in both ECs and ISCs. Its activation in ISCs induces their proliferation by phosphorylating the AP-1 transcription factor Fos (Biteau et al., 2008; Biteau and Jasper, 2011; Hochmuth et al., 2011). Interestingly, Fos is phosphorylated both by JNK and EGFR pathways, and thus integrates growth factor and stress signals to induce ISC proliferation (Ciapponi et al., 2001; Biteau and Jasper, 2011). JNK activation in ECs, on the other hand, can stimulate Upd induction and induce ISC proliferation, but does not seem to be required for the regenerative response to a challenge (Jiang et al., 2009) nor for survival of the host upon pathogenic infection (Buchon et al., 2009a). Forced activation of JNK in ECs induces Upd expression by promoting Yorkie nuclear localization (Karpowicz et al., 2010; Ren et al., 2010; Shaw et al., 2010; Staley and Irvine, 2010).

The need for epithelial renewal after pathogenic infections suggest that to maintain homeostasis, signaling mechanisms that control innate immune and inflammatory responses and signaling pathways that regulate ISC proliferation have to be highly coordinated. Recent years have seen tremendous progress in our understanding of the cellular and molecular mechanisms governing this coordination (Buchon et al., 2013a).

## Intestinal immunity

The *Drosophila* intestine contains physical and chemical barriers to prevent microbial infections [reviewed in Ferrandon (2013)]. As a first barrier, a peritrophic matrix, consisting of chitin and glycoproteins covers the intestinal epithelium, preventing direct contact of microbes and other lumen contents with epithelial cells. The peritrophic matrix is secreted by the proventriculus with possible contributions by ECs (Hegedus et al., 2009). Loss of peritrophic matrix components renders flies susceptible to infections, highlighting the importance of the peritrophic matrix as a physical barrier against bacteria (Kuraishi et al., 2011).

A second line of defense is the secretion of antimicrobial peptides (AMPs) by ECs in the intestinal epithelium (Tzou et al., 2000; Liehl et al., 2006; Ryu et al., 2006; Nehme et al., 2007; Buchon et al., 2009b). Invading bacteria are recognized by their peptidoglycans (PGN; structural components of the bacterial cell wall) (Zaidman-Remy et al., 2006). PGNs bind to PGRP-LC and -LE resulting in activation of the IMD/Relish (but not the Toll) pathway (Bosco-Drayon et al., 2012; Neyen et al., 2012), which in turn induces AMP transcription [immune signaling in *D. melanogaster* is comprehensively reviewed in Ferrandon et al. (2007); Lemaitre and Hoffmann (2007); Ha et al. (2009a); Davis and Engstrom (2012); Buchon et al. (2013a); Lee and Brey (2013)]. Relish belongs to the family of highly conserved Nuclear Factor-κB (NF-κB) transcription factors, and is a required component of the IMD pathway, which is related to the mammalian tumor necrosis factor receptor (TNFR) pathway (Hoffmann, 2003). NF-κB and TNFR pathways are critical for epithelial immunity in mammals (Xavier and Podolsky, 2007; Meylan et al., 2011; De Jong et al., 2012): NF-κB activation in epithelial cells modulates immune responses to environmental challenges and microbial infections (Pasparakis, 2012), and cytokines and chemokines secreted by epithelial cells act on immune and non-immune cells to modulate the cellular immune response. Chronic activation of NF-κB and of the TNFR pathway in epithelial cells results in the development of intestinal inflammation (Meylan et al., 2011; Wullaert et al., 2011; De Jong et al., 2012).

**Figure 2:**
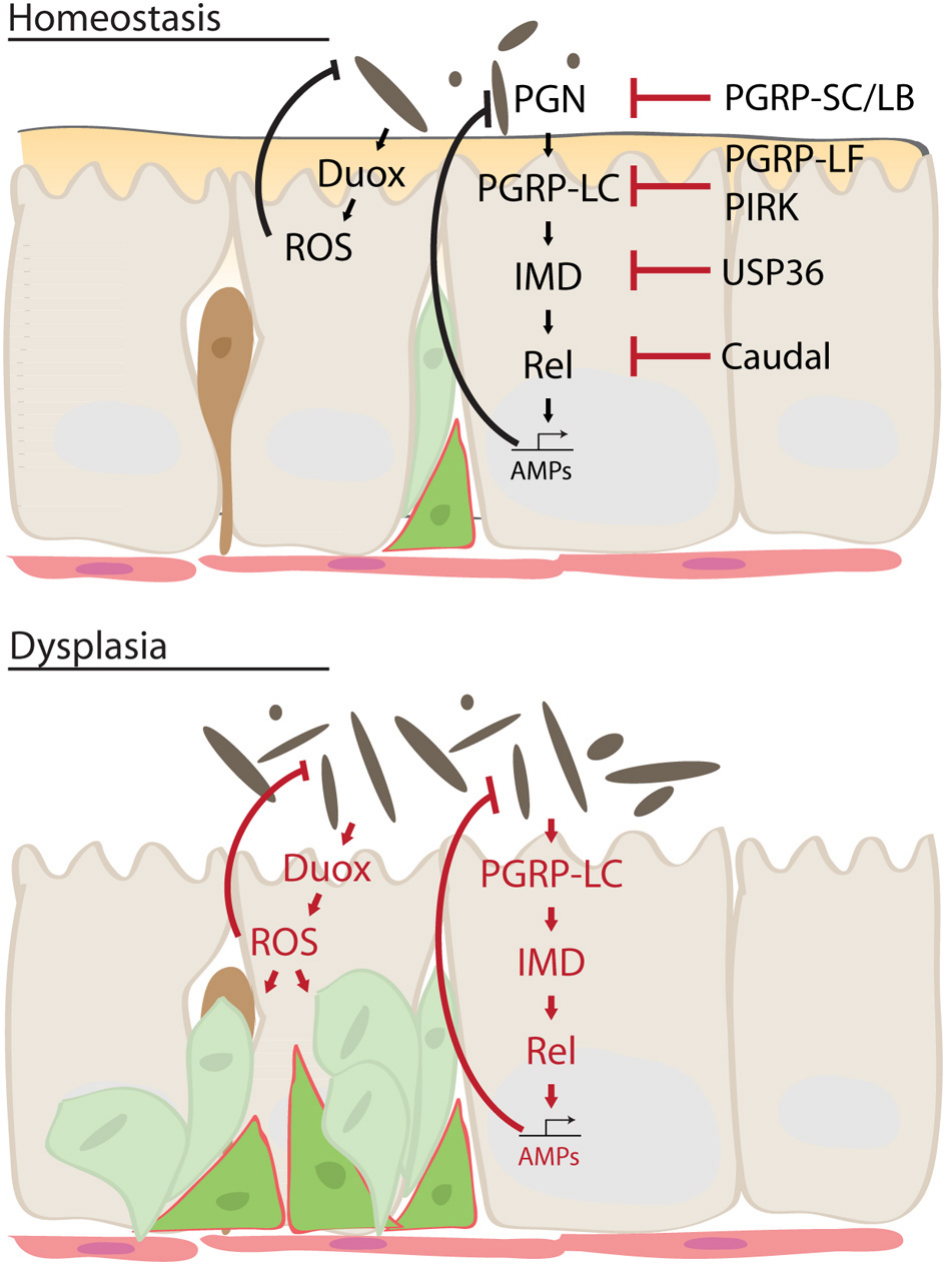
**Mechanism of intestinal dysplasia**. Under normal homeostatic conditions, activity of Immune deficiency (IMD) pathway is tightly regulated by multiple factors. This ensures moderate innate immune response sufficient to keep intestinal microbes in check while preventing excessive immune activation. In an aging intestine, however, loss of these regulatory mechanisms leads to chronic inflammation and dysbiosis, which results in dysplasia and disruption of tissue homeostasis. Dual oxidase (Duox), reactive oxygen species (ROS), peptidoglycan (PGRP), Relish (Rel), poor Imd response upon knock-in (PIRK), antimicrobial peptides (AMP).

In flies, the IMD/Rel pathway is kept inactive in normal, homeostatic, conditions by a variety of negative regulators, including Caudal (Ryu et al., 2008), PGRPs of the SC, LB and LF class (Zaidman-Remy et al., 2006; Maillet et al., 2008; Paredes et al., 2011), USP36 (Thevenon et al., 2009) and PIRK (Lhocine et al., 2008) (Figure 2). These regulators are of particular importance in the maintenance of not only the commensal population, but also of proliferative homeostasis in the intestinal epithelium: loss of the homeobox transcription factor Caudal, for example, leads to a shift in commensal populations in the fly intestine, eliminating beneficial bacterial species and allowing outbreaks of pathogenic species. At the same time, stress signaling is ectopically activated, and stem cell proliferation is strongly induced, resulting in dysplasia-like phenotypes (Ryu et al., 2008; Buchon et al., 2009a; Biteau et al., 2010). These conditions are reminiscent of the dysplasia and inflammation observed in aging flies, where microbial expansion is associated with hyperactivation of the IMD, JAK/Stat and JNK signaling pathways, and with epithelial dysplasia (Buchon et al., 2009a).

The third part of the intestinal immune response against microbes is the production of ROS by ECs. ROS are produced by the transmembrane protein dual oxidase (DUOX), a member of the NADPH oxidase family, which is transcriptionally induced in ECs and activated in response to a bacterial challenge (Ha et al., 2005; Ryu et al., 2010). Under homeostatic conditions, ROS are produced at moderate levels in response to the interaction of the epithelium with resident autochthonous bacterial species. During infection with transient allochthonous bacteria, however, production of ROS is increased by two mechanisms: an unknown G-Protein Coupled Receptor (GPCR) activates Phospholipase C-β (PLCβ) and triggers inositol-1,4,5-triphosphate (IP3)-induced Ca2+ release. Ca2+ is bound by EF-hands in DUOX, stimulating its activity (Ha et al., 2009a). A second mechanism involves activation of p38 MAPKinase, which transcriptionally induces Duox (Ha et al., 2009a,b). Young flies are believed to protect themselves from the cytotoxic effects of ROS by secreting an extracellular immune-related catalase (IRC), which neutralizes ROS (Ha et al., 2005). However, excessive ROS are generated and accumulate in the intestine of aged flies, presumably as a consequence of constant stimulation by immune resistant intestinal microbes. This excessive ROS accumulation is a likely cause of the age-related loss of epithelial homeostasis (Buchon et al., 2009a; Hochmuth et al., 2011).

## Intestinal microbiota

A young and healthy *Drosophila* intestine contains a relatively simple microbiota comprising about 5–20 microbial species. Major constituents of these commensals are beneficial microbes, such as *Acetobacter pomorum* and *Lactobacillus plantarum* which promote growth and development in flies when reared on a restricted diet (Ryu et al., 2008; Chandler et al., 2011; Shin et al., 2011; Storelli et al., 2011; Wong et al., 2011). These microbes do not activate the intestinal immune system, allowing colonization of the gut. Resident pathobionts or invading potential pathogens, on the other hand, are readily recognized. One example of such a pathobiont, *Gluconobacter morbifer*, constitutes only a minor proportion of the healthy intestinal community. Under favorable conditions, however, it can take over the gut, causing gut pathologies and lethality of the host (Ryu et al., 2008). Moreover, many negative regulators have also been identified that prevent chronic activation of the IMD pathway induced by indigenous microbes (Lhocine et al., 2008; Paredes et al., 2011; Bosco-Drayon et al., 2012).

Until recently, it was not known how the *Drosophila* immune system differentiates between friends and foes. Recent elegant work by the Lee lab shows, however, that pathogenic bacteria, in contrast to beneficial symbionts, are constantly secreting Uracil, which is recognized by the *Drosophila* immune system. Uracil is recognized by an unknown GPCR, which activates the PLCb/IP3/Ca/Duox pathway discussed above to produce ROS. Many opportunistic pathogens such as *Vibrio fluvialis, Klebsiella pneumonia, Erwinia carotovora carotovora, Shigella sonnei, Pseudomonas aeruginosa* and *Serratia marcescens*, but not symbionts like *A. pomorum*, *L. plantarum* and *Commensalibacter intestini*, secrete significant quantities of Uracil (Lee et al., 2013). Mono-association of flies with *G. morbifer* leads to chronic inflammation, induces apoptosis and shortens *Drosophila* lifespan, while these effects were not observed in germ free control flies or in flies mono-associated with a mutant *G. morbifer* lacking Uracil secretion. Uracil-producing pathobionts may also contribute significantly to the age-related increase in epithelial Duox-mediated ROS production, and thus to the age-associated dysfunction in epithelial homeostasis. It is clear that understanding causes and consequences of age-related changes in the commensal microbiome is an important task for future studies.

## Inflammation regeneration crosstalk

The Duox-induced innate immune response has thus important implications for the etiology of the dysfunction of the intestinal epithelium observed in aging flies. When flies are raised on a conventional diet, internal and external microbial populations expand with age (Ren et al., 2007; Guo et al., in press) and this expansion correlates with the age-associated accumulation of ROS in the gut (Buchon et al., 2009a). The increasing concentration of ROS stimulates ISC proliferation directly by activating JNK or inhibiting the Nrf2 homolog CncC (Biteau et al., 2008; Hochmuth et al., 2011), or indirectly by damaging ECs and stimulating Upd expression (Jiang et al., 2009). Although increased ISC activity is essential for regeneration in young epithelia in response to an insult, excessive ISC proliferation in aging animals results in the accumulation of mis-differentiated cells and the loss of tissue homeostasis, and is thus deleterious to animal health (Biteau et al., 2008, 2010; Hochmuth et al., 2011). Accordingly, overexpressing antioxidants or other stress-protective factors within ISCs not only rescues this age-related dysplasia, but also extends lifespan in *Drosophila* (Biteau et al., 2010; Hochmuth et al., 2011; Rera et al., 2011). These observations further support the notion that age-associated changes in the intestinal microbiota play a critical role in the development of age-related pathologies of the intestine, a concept that further studies in the fly should be able to test.

Why does the commensal microbiota expand in aging animals? It is unclear whether malfunctioning of the host immune response causes commensal populations to overgrow, or if expansion of immune-resistant intestinal commensals is the initiating event that causes the described aged-related intestinal pathology. Deregulation of innate immune signaling in aging epithelia can be observed, and may be brought about by age-related activation of stress signaling, in particular of the JNK signaling pathway (Buchon et al., 2009a; Karpac et al., 2009, 2013). The interaction between the IMD pathway and JNK is multifactorial and complex: JNK-mediated activation of the transcription factor Foxo can induce Rel expression in larvae (Karpac et al., 2011). In larval fatbodies, activation of TAK1 by infection not only promotes Relish nuclear localization, but also activates Hemipterous (JNKK), which phosphorylates and activates Basket (JNK) (Silverman et al., 2003; Park et al., 2004; Kallio et al., 2005). Another IMD pathway component, DREDD, may also directly activate JNK upon immune stimulation (Guntermann and Foley, 2011). Furthermore, JNK and Foxo have been shown to induce AMP transcription, in part independently of Relish (Delaney et al., 2006; Becker et al., 2010). Recent work in larvae and adults highlights the need to study these interactions in a spatially- and temporally-resolved manner in order to characterize the complex interactions between innate immune responses and stress and inflammatory signals *in vivo* (Karpac et al., 2011, 2013). Interestingly, a recent study from our lab identified age-related activation of Foxo in ECs as a driving force in the disruption of innate immune homeostasis, resulting in immune senescence. Foxo inhibits the expression of PGRP-SC2, resulting in chronic, excessive activation of Relish, and impairing the ability of the intestine to clear bacteria (Guo et al., in press).

While germ-free conditions can rescue age-related dysplasia (Buchon et al., 2009a), and pharmacological inhibition of the NFκB signaling pathway can reportedly extend *Drosophila* lifespan (Moskalev and Shaposhnikov, 2011), the evidence for a role of intestinal microbiota in influencing fly longevity remains controversial (Brummel et al., 2004; Ren et al., 2007). It is likely that rearing flies under sterile conditions results in the removal of not only deleterious species, but also of beneficial commensals, and experiments assessing fly lifespan under germ-free conditions may thus result in variable outcomes. However, moderate downregulation of ISC proliferation has been shown to not only rescue the age-related intestinal disruption but also to extend lifespan (Biteau et al., 2010; Hochmuth et al., 2011). Conditions that can keep the commensal bacterial population in check, promoting innate immune homeostasis and proliferative homeostasis in the intestinal epithelium, are thus expected to be beneficial for the animal's health. Accordingly, we find that managing the commensal population by preventing the age-related loss of PGRP-SC2 expression is sufficient to limit age-related dysplasia and extend lifespan (Guo et al., in press).

## Conclusion

To maintain intestinal homeostasis, a highly selective immune response has to ensure that pathogenic microorganisms are eliminated, while commensals can thrive. Moreover, the inflammatory response triggered by pathogens and commensals alike has to be carefully contained to prevent excessive stem cell activation and dysplasias. It may not be surprising that these carefully balanced responses are misregulated in aging animals, making the host more susceptible to invading microbes, and promoting inflammatory and dysplastic conditions. A better understanding of the molecular parameters driving these age-related changes, however, promises to provide insight into avenues for therapeutic intervention that may not only be applied to inflammatory diseases and cancers of the gut, but potentially to allay tissue dysfunction in the normally aging human intestine.

### Conflict of interest statement

The authors declare that the research was conducted in the absence of any commercial or financial relationships that could be construed as a potential conflict of interest.

## References

[B1] Amcheslavsky A. Ito N. Jiang J. Ip Y. T. (2011). Tuberous sclerosis complex and Myc coordinate the growth and division of Drosophila intestinal stem cells. J. Cell Biol. 193, 695–710 10.1083/jcb.201103018 21555458 PMC3166862

[B2] Apidianakis Y. Pitsouli C. Perrimon N. Rahme L. (2009). Synergy between bacterial infection and genetic predisposition in intestinal dysplasia. Proc. Natl. Acad. Sci. U.S.A. 106, 20883–20888 10.1073/pnas.0911797106 19934041 PMC2791635

[B3] Apidianakis Y. Rahme L. G. (2011). *Drosophila melanogaster* as a model for human intestinal infection and pathology. Dis. Model. Mech. 4, 21–30 10.1242/dmm.003970 21183483 PMC3014343

[B4] Bardin A. J. Perdigoto C. N. Southall T. D. Brand A. H. Schweisguth F. (2010). Transcriptional control of stem cell maintenance in the Drosophila intestine. Development 137, 705–714 10.1242/dev.039404 20147375 PMC2827683

[B5] Becker T. Loch G. Beyer M. Zinke I. Aschenbrenner A. C. Carrera P. (2010). FOXO-dependent regulation of innate immune homeostasis. Nature 463, 369–373 10.1038/nature08698 20090753

[B6] Biagi E. Candela M. Fairweather-Tait S. Franceschi C. Brigidi P. (2012). Aging of the human metaorganism: the microbial counterpart. Age (Dordr.) 34, 247–267 10.1007/s11357-011-9217-5 21347607 PMC3260362

[B7] Biagi E. Nylund L. Candela M. Ostan R. Bucci L. Pini E. (2010). Through ageing, and beyond: gut microbiota and inflammatory status in seniors and centenarians. PLoS ONE 5:e10667 10.1371/journal.pone.0010667 20498852 PMC2871786

[B8] Biteau B. Hochmuth C. E. Jasper H. (2008). JNK activity in somatic stem cells causes loss of tissue homeostasis in the aging Drosophila gut. Cell Stem Cell 3, 442–455 10.1016/j.stem.2008.07.024 18940735 PMC3225008

[B9] Biteau B. Hochmuth C. E. Jasper H. (2011a). Maintaining tissue homeostasis: dynamic control of somatic stem cell activity. Cell Stem Cell 9, 402–411 10.1016/j.stem.2011.10.004 22056138 PMC3212030

[B10] Biteau B. Karpac J. Hwangbo D. Jasper H. (2011b). Regulation of Drosophila lifespan by JNK signaling. Exp. Gerontol. 46, 349–354 10.1016/j.exger.2010.11.003 21111799 PMC3079798

[B11] Biteau B. Jasper H. (2011). EGF signaling regulates the proliferation of intestinal stem cells in Drosophila. Development 138, 1045–1055 10.1242/dev.056671 21307097 PMC3042864

[B12] Biteau B. Karpac J. Supoyo S. Degennaro M. Lehmann R. Jasper H. (2010). Lifespan extension by preserving proliferative homeostasis in Drosophila. PLoS Genet. 6:e1001159 10.1371/journal.pgen.1001159 20976250 PMC2954830

[B13] Bosco-Drayon V. Poidevin M. Boneca I. G. Narbonne-Reveau K. Royet J. Charroux B. (2012). Peptidoglycan sensing by the receptor PGRP-LE in the Drosophila gut induces immune responses to infectious bacteria and tolerance to microbiota. Cell Host Microbe 12, 153–165 10.1016/j.chom.2012.06.002 22901536

[B14] Brummel T. Ching A. Seroude L. Simon A. F. Benzer S. (2004). Drosophila lifespan enhancement by exogenous bacteria. Proc. Natl. Acad. Sci. U.S.A. 101, 12974–12979 10.1073/pnas.0405207101 15322271 PMC516503

[B15] Buchon N. Broderick N. A. Chakrabarti S. Lemaitre B. (2009a). Invasive and indigenous microbiota impact intestinal stem cell activity through multiple pathways in Drosophila. Genes Dev. 23, 2333–2344 10.1101/gad.1827009 19797770 PMC2758745

[B16] Buchon N. Broderick N. A. Poidevin M. Pradervand S. Lemaitre B. (2009b). Drosophila intestinal response to bacterial infection: activation of host defense and stem cell proliferation. Cell Host Microbe 5, 200–211 10.1016/j.chom.2009.01.003 19218090

[B17] Buchon N. Broderick N. A. Kuraishi T. Lemaitre B. (2010). Drosophila EGFR pathway coordinates stem cell proliferation and gut remodeling following infection. BMC Biol. 8:152 10.1186/1741-7007-8-152 21176204 PMC3022776

[B18] Buchon N. Broderick N. A. Lemaitre B. (2013a). Gut homeostasis in a microbial world: insights from *Drosophila melanogaster*. Nat. Rev. Microbiol. 11, 615–626 10.1038/nrmicro3074 23893105

[B19] Buchon N. Osman D. David F. P. A. Fang H. Y. Boquete J.-P. Deplancke B. (2013b). Morphological and molecular characterization of adult midgut compartmentalization in Drosophila. Cell Rep. 3, 1725–1738 10.1016/j.celrep.2013.04.001 23643535

[B20] Chandler J. A. Lang J. M. Bhatnagar S. Eisen J. A. Kopp A. (2011). Bacterial communities of diverse Drosophila species: ecological context of a host-microbe model system. PLoS Genet. 7:e1002272 10.1371/journal.pgen.1002272 21966276 PMC3178584

[B21] Choi N.-H. Kim J.-G. Yang D.-J. Kim Y.-S. Yoo M.-A. (2008). Age-related changes in Drosophila midgut are associated with PVF2, a PDGF/VEGF-like growth factor. Aging Cell 7, 318–334 10.1111/j.1474-9726.2008.00380.x 18284659 PMC2408640

[B22] Ciapponi L. Jackson D. B. Mlodzik M. Bohmann D. (2001). Drosophila Fos mediates ERK and JNK signals via distinct phosphorylation sites. Genes Dev. 15, 1540–1553 10.1101/gad.886301 11410534 PMC312716

[B23] Claesson M. J. Cusack S. O'sullivan O. Greene-Diniz R. De Weerd H. Flannery E. (2011). Composition, variability, and temporal stability of the intestinal microbiota of the elderly. Proc. Natl. Acad. Sci. U.S.A. 108(Suppl.) 4586–4591 10.1073/pnas.1000097107 20571116 PMC3063589

[B24] Claesson M. J. Jeffery I. B. Conde S. Power S. E. O'connor E. M. Cusack S. (2012). Gut microbiota composition correlates with diet and health in the elderly. Nature 488, 178–184 10.1038/nature11319 22797518

[B25] Clemente J. C. Ursell L. K. Parfrey L. W. Knight R. (2012). The impact of the gut microbiota on human health: an integrative view. Cell 148, 1258–1270 10.1016/j.cell.2012.01.035 22424233 PMC5050011

[B26] Cordero J. B. Sansom O. J. (2012). Wnt signalling and its role in stem cell-driven intestinal regeneration and hyperplasia. Acta Physiol. (Oxf.) 204, 137–143 10.1111/j.1748-1716.2011.02288.x 21439026

[B27] Cordero J. B. Stefanatos R. K. Myant K. Vidal M. Sansom O. J. (2012a). Non-autonomous crosstalk between the Jak/Stat and Egfr pathways mediates Apc1-driven intestinal stem cell hyperplasia in the Drosophila adult midgut. Development 139, 4524–4535 10.1242/dev.078261 23172913

[B28] Cordero J. B. Stefanatos R. K. Scopelliti A. Vidal M. Sansom O. J. (2012b). Inducible progenitor-derived Wingless regulates adult midgut regeneration in Drosophila. EMBO J. 31, 3901–3917 10.1038/emboj.2012.248 22948071 PMC3463851

[B29] Cronin S. J. F. Nehme N. T. Limmer S. Liegeois S. Pospisilik J. A. Schramek D. (2009). Genome-wide RNAi screen identifies genes involved in intestinal pathogenic bacterial infection. Science 325, 340–343 10.1126/science.1173164 19520911 PMC2975362

[B30] Davis M. M. Engstrom Y. (2012). Immune response in the barrier epithelia: lessons from the fruit fly *Drosophila melanogaster*. J. Innate Immun. 4, 273–283 10.1159/000332947 22237424 PMC6741545

[B31] De Bandt J.-P. Waligora-Dupriet A.-J. Butel M.-J. (2011). Intestinal microbiota in inflammation and insulin resistance: relevance to humans. Curr. Opin. Clin. Nutr. Metab. Care 14, 334–340 10.1097/MCO.0b013e328347924a 21587065

[B32] De Jong H. K. Parry C. M. Van Der Poll T. Wiersinga W. J. (2012). Host-pathogen interaction in invasive Salmonellosis. PLoS Pathog. 8:e1002933 10.1371/journal.ppat.1002933 23055923 PMC3464234

[B33] Delaney J. R. Stöven S. Uvell H. Anderson K. V. Engström Y. Mlodzik M. (2006). Cooperative control of Drosophila immune responses by the JNK and NF-kappaB signaling pathways. EMBO J. 25, 3068–3077 10.1038/sj.emboj.7601182 16763552 PMC1500970

[B34] Duncan S. H. Flint H. J. (2013). Probiotics and prebiotics and health in ageing populations. Maturitas 75, 44–50 10.1016/j.maturitas.2013.02.004 23489554

[B35] Ferrandon D. (2013). The complementary facets of epithelial host defenses in the genetic model organism *Drosophila melanogaster*: from resistance to resilience. Curr. Opin. Immunol. 25, 59–70 10.1016/j.coi.2012.11.008 23228366

[B36] Ferrandon D. Imler J.-L. Hetru C. Hoffmann J. A. (2007). The Drosophila systemic immune response: sensing and signalling during bacterial and fungal infections. Nat. Rev. Immunol. 7, 862–874 10.1038/nri2194 17948019

[B37] Gonda T. A. Tu S. Wang T. C. (2009). Chronic inflammation, the tumor microenvironment and carcinogenesis. Cell cycle 8, 2005–2013 10.4161/cc.8.13.8985 19550141

[B38] Goulas S. Conder R. Knoblich J. A. (2012). The Par complex and integrins direct asymmetric cell division in adult intestinal stem cells. Cell Stem Cell 11, 529–540 10.1016/j.stem.2012.06.017 23040479 PMC3465556

[B39] Guntermann S. Foley E. (2011). The protein Dredd is an essential component of the c-Jun N-terminal kinase pathway in the Drosophila immune response. J. Biol. Chem. 286, 30284–30294 10.1074/jbc.M111.220285 21730059 PMC3162387

[B40] Guo L. Karpac J. Tran S. L. Jasper H. (in press). Lifespan extension by promoting immune homeostasis and limiting commensal dysbiosis in the Drosophila intestine. Cell.

[B41] Guo Z. Driver I. Ohlstein B. (2013). Injury-induced BMP signaling negatively regulates Drosophila midgut homeostasis. J. Cell Biol. 201, 945–961 10.1083/jcb.201302049 23733344 PMC3678160

[B42] Ha E. M. Oh C. T. Bae Y. S. Lee W. J. (2005). A direct role for dual oxidase in Drosophila gut immunity. Science 310, 847–850 10.1126/science.1117311 16272120

[B43] Ha E. M. Lee K. A. Park S. H. Kim S. H. Nam H. J. Lee H. Y. (2009a). Regulation of DUOX by the Galphaq-phospholipase Cbeta-Ca2+ pathway in Drosophila gut immunity. Dev. Cell 16, 386–397 10.1016/j.devcel.2008.12.015 19289084

[B44] Ha E. M. Lee K. A. Seo Y. Y. Kim S. H. Lim J. H. Oh B. H. (2009b). Coordination of multiple dual oxidase-regulatory pathways in responses to commensal and infectious microbes in drosophila gut. Nat. Immunol. 10, 949–957 10.1038/ni.1765 19668222

[B45] Hegedus D. Erlandson M. Gillott C. Toprak U. (2009). New insights into peritrophic matrix synthesis, architecture, and function. Annu. Rev. Entomol. 54, 285–302 10.1146/annurev.ento.54.110807.090559 19067633

[B46] Hochmuth C. E. Biteau B. Bohmann D. Jasper H. (2011). Redox regulation by Keap1 and Nrf2 controls intestinal stem cell proliferation in Drosophila. Cell Stem Cell 8, 188–199 10.1016/j.stem.2010.12.006 21295275 PMC3035938

[B47] Hoffmann J. A. (2003). The immune response of Drosophila. Nature 426, 33–38 10.1038/nature02021 14603309

[B48] Jiang H. Edgar B. A. (2009). EGFR signaling regulates the proliferation of Drosophila adult midgut progenitors. Development 136, 483–493 10.1242/dev.026955 19141677 PMC2687592

[B49] Jiang H. Patel P. H. Kohlmaier A. Grenley M. O. Mcewen D. G. Edgar B. A. (2009). Cytokine/Jak/Stat signaling mediates regeneration and homeostasis in the Drosophila midgut. Cell 137, 1343–1355 10.1016/j.cell.2009.05.014 19563763 PMC2753793

[B50] Kallio J. Leinonen A. Ulvila J. Valanne S. Ezekowitz R. A. Rämet M. (2005). Functional analysis of immune response genes in Drosophila identifies JNK pathway as a regulator of antimicrobial peptide gene expression in S2 cells. Microbes Infect. 7, 811–819 10.1016/j.micinf.2005.03.014 15890554

[B51] Kallus S. J. Brandt L. J. (2012). The intestinal microbiota and obesity. J. Clin. Gastroenterol. 46, 16–24 10.1097/MCG.0b013e31823711fd 22064556

[B52] Kamada N. Seo S.-U. Chen G. Y. Núñez G. (2013). Role of the gut microbiota in immunity and inflammatory disease. Nat. Rev. Immunol. 13, 321–335 10.1038/nri3430 23618829

[B53] Kapuria S. Karpac J. Biteau B. Hwangbo D. Jasper H. (2012). Notch-mediated suppression of TSC2 expression regulates cell differentiation in the Drosophila intestinal stem cell lineage. PLoS Genet. 8:e1003045 10.1371/journal.pgen.1003045 23144631 PMC3493453

[B54] Karpac J. Biteau B. Jasper H. (2013). Misregulation of an adaptive metabolic response contributes to the age-related disruption of lipid homeostasis in Drosophila. Cell Rep. 4, 1250–1261 10.1016/j.celrep.2013.08.004 24035390 PMC3832190

[B55] Karpac J. Hull-Thompson J. Falleur M. Jasper H. (2009). JNK signaling in insulin-producing cells is required for adaptive responses to stress in Drosophila. Aging Cell 8, 288–295 10.1111/j.1474-9726.2009.00476.x 19627268 PMC2727449

[B56] Karpac J. Jasper H. (2009). Insulin and JNK: optimizing metabolic homeostasis and lifespan. Trends Endocrinol. Metab. 20, 100–106 10.1016/j.tem.2008.11.004 19251431 PMC3227503

[B57] Karpac J. Younger A. Jasper H. (2011). Dynamic coordination of innate immune signaling and insulin signaling regulates systemic responses to localized DNA damage. Dev. Cell 20, 841–854 10.1016/j.devcel.2011.05.011 21664581 PMC3151532

[B58] Karpowicz P. Perez J. Perrimon N. (2010). The Hippo tumor suppressor pathway regulates intestinal stem cell regeneration. Development 137, 4135–4145 10.1242/dev.060483 21098564 PMC2990205

[B59] Kaser A. Zeissig S. Blumberg R. S. (2010). Inflammatory bowel disease. Annu. Rev. Immunol. 28, 573–621 10.1146/annurev-immunol-030409-101225 20192811 PMC4620040

[B60] Kenyon C. J. (2010). The genetics of ageing. Nature 464, 504–512 10.1038/nature08980 20336132

[B61] Koeth R. A. Wang Z. Levison B. S. Buffa J. A. Org E. Sheehy B. T. (2013). Intestinal microbiota metabolism of L-carnitine, a nutrient in red meat, promotes atherosclerosis. Nat. Med. 19, 576–585 10.1038/nm.3145 23563705 PMC3650111

[B62] Kostic A. D. Gevers D. Pedamallu C. S. Michaud M. Duke F. Earl A. M. (2012). Genomic analysis identifies association of Fusobacterium with colorectal carcinoma. Genome Res. 22, 292–298 10.1101/gr.126573.111 22009990 PMC3266036

[B63] Kuraishi T. Binggeli O. Opota O. Buchon N. Lemaitre B. (2011). Genetic evidence for a protective role of the peritrophic matrix against intestinal bacterial infection in *Drosophila melanogaster*. Proc. Natl. Acad. Sci. U.S.A. 108, 15966–15971 10.1073/pnas.1105994108 21896728 PMC3179054

[B64] Lee K.-A. Kim S.-H. Kim E.-K. Ha E.-M. You H. Kim B. (2013). Bacterial-derived uracil as a modulator of mucosal immunity and gut-microbe homeostasis in Drosophila. Cell 153, 797–811 10.1016/j.cell.2013.04.009 23663779

[B65] Lee W.-J. Brey P. T. (2013). How microbiomes influence metazoan development: insights from history and Drosophila modeling of gut-microbe interactions. Annu. Rev. Cell Dev. Boil. 29, 571–592 10.1146/annurev-cellbio-101512-122333 23808845

[B66] Lemaitre B. Hoffmann J. (2007). The host defense of *Drosophila melanogaster*. Annu. Rev. Immunol. 25, 697–743 10.1146/annurev.immunol.25.022106.141615 17201680

[B67] Lemaitre B. Miguel-Aliaga I. (2013). The digestive tract of *Drosophila melanogaster.* Annu. Rev. Genet. 47, 377–404 10.1146/annurev-genet-111212-133343 24016187

[B68] Lhocine N. Ribeiro P. S. Buchon N. Wepf A. Wilson R. Tenev T. (2008). PIMS modulates immune tolerance by negatively regulating Drosophila innate immune signaling. Cell Host Microbe 4, 147–158 10.1016/j.chom.2008.07.004 18692774

[B69] Li H. Qi Y. Jasper H. (2013). Dpp signaling determines regional stem cell identity in the regenerating adult Drosophila gastrointestinal tract. Cell Rep. 4, 10–18 10.1016/j.celrep.2013.05.040 23810561 PMC3778028

[B70] Liehl P. Blight M. Vodovar N. Boccard F. Lemaitre B. (2006). Prevalence of local immune response against oral infection in a Drosophila/Pseudomonas infection model. PLoS Pathog. 2:e56 10.1371/journal.ppat.0020056 16789834 PMC1475658

[B71] Lin G. Xu N. Xi R. (2008). Paracrine wingless signalling controls self-renewal of Drosophila intestinal stem cells. Nature 455, 1119–1123 10.1038/nature07329 18806781

[B72] Lozupone C. A. Stombaugh J. I. Gordon J. I. Jansson J. K. Knight R. (2012). Diversity, stability and resilience of the human gut microbiota. Nature 489, 220–230 10.1038/nature11550 22972295 PMC3577372

[B73] Maillet F. Bischoff V. Vignal C. Hoffmann J. Royet J. (2008). The Drosophila peptidoglycan recognition protein PGRP-LF blocks PGRP-LC and IMD/JNK pathway activation. Cell Host Microbe 3, 293–303 10.1016/j.chom.2008.04.002 18474356

[B74] Marianes A. Spradling A. C. (2013). Physiological and stem cell compartmentalization within the Drosophila midgut. eLife 2:e00886 10.7554/eLife.00886 23991285 PMC3755342

[B75] Meylan F. Richard A. C. Siegel R. M. (2011). TL1A and DR3, a TNF family ligand-receptor pair that promotes lymphocyte costimulation, mucosal hyperplasia, and autoimmune inflammation. Immunol. Rev. 244, 188–196 10.1111/j.1600-065X.2011.01068.x 22017439 PMC3882070

[B76] Micchelli C. A. Perrimon N. (2006). Evidence that stem cells reside in the adult Drosophila midgut epithelium. Nature 439, 475–479 10.1038/nature04371 16340959

[B77] Moskalev A. Shaposhnikov M. (2011). Pharmacological inhibition of NF-κB prolongs lifespan of *Drosophila melanogaster*. Aging 3, 391–394 21483034 10.18632/aging.100314 PMC3117454

[B78] Nehme N. T. Liégeois S. Kele B. Giammarinaro P. Pradel E. Hoffmann J. A. (2007). A model of bacterial intestinal infections in *Drosophila melanogaster*. PLoS Pathog. 3:e173 10.1371/journal.ppat.0030173 18039029 PMC2094306

[B79] Neyen C. Poidevin M. Roussel A. Lemaitre B. (2012). Tissue- and ligand-specific sensing of gram-negative infection in drosophila by PGRP-LC isoforms and PGRP-LE. J. Immunol. 189, 1886–1897 10.4049/jimmunol.1201022 22772451

[B80] Niwa T. Tsukamoto T. Toyoda T. Mori A. Tanaka H. Maekita T. (2010). Inflammatory processes triggered by *Helicobacter pylori* infection cause aberrant DNA methylation in gastric epithelial cells. Cancer Res. 70, 1430–1440 10.1158/0008-5472.CAN-09-2755 20124475

[B81] O'brien L. E. Soliman S. S. Li X. Bilder D. (2011). Altered modes of stem cell division drive adaptive intestinal growth. Cell 147, 603–614 10.1016/j.cell.2011.08.048 22036568 PMC3246009

[B82] Ohlstein B. Spradling A. (2006). The adult Drosophila posterior midgut is maintained by pluripotent stem cells. Nature 439, 470–474 10.1038/nature04333 16340960

[B83] Ohlstein B. Spradling A. (2007). Multipotent Drosophila intestinal stem cells specify daughter cell fates by differential notch signaling. Science 315, 988–992 10.1126/science.1136606 17303754

[B84] Osman D. Buchon N. Chakrabarti S. Huang Y. T. Su W. C. Poidevin M. (2012). Autocrine and paracrine unpaired signaling regulate intestinal stem cell maintenance and division. J. Cell Sci. 125, 5944–5949 10.1242/jcs.113100 23038775

[B85] Paaby A. B. Schmidt P. S. (2008). Functional significance of allelic variation at methuselah, an aging gene in Drosophila. PLoS ONE 3:e1987 10.1371/journal.pone.0001987 18414670 PMC2288678

[B86] Paredes J. C. Welchman D. P. Poidevin M. Lemaitre B. (2011). Negative regulation by amidase PGRPs shapes the Drosophila antibacterial response and protects the fly from innocuous infection. Immunity 35, 770–779 10.1016/j.immuni.2011.09.018 22118526

[B87] Park J. M. Brady H. Ruocco M. G. Sun H. Williams D. Lee S. J. (2004). Targeting of TAK1 by the NF-kappa B protein Relish regulates the JNK-mediated immune response in Drosophila. Genes Dev. 18, 584–594 10.1101/gad.1168104 15037551 PMC374239

[B88] Park J.-S. Kim Y.-S. Yoo M.-A. (2009). The role of p38b MAPK in age-related modulation of intestinal stem cell proliferation and differentiation in Drosophila. Aging 1, 637–651 20157545 10.18632/aging.100054 PMC2806044

[B89] Pasparakis M. (2012). Role of NF-κ B in epithelial biology. Immunol. Rev. 246, 346–358 10.1111/j.1600-065X.2012.01109.x 22435565

[B90] Patel B. B. Yu Y. Du J. Levi E. Phillip P. A. Majumdar A. P. N. (2009). Age-related increase in colorectal cancer stem cells in macroscopically normal mucosa of patients with adenomas: a risk factor for colon cancer. Biochem. Biophys. Res. Commun. 378, 344–347 10.1016/j.bbrc.2008.10.179 19010307 PMC2644999

[B91] Perdigoto C. N. Schweisguth F. Bardin A. J. (2011). Distinct levels of Notch activity for commitment and terminal differentiation of stem cells in the adult fly intestine. Development 138, 4585–4595 10.1242/dev.065292 21965616

[B92] Power S. E. O'toole P. W. Stanton C. Ross R. P. Fitzgerald G. F. (2013). Intestinal microbiota, diet and health. Br. J. Nutr. 1–16 10.1017/S0007114513002560 23931069

[B93] Qin J. Li R. Raes J. Arumugam M. Burgdorf K. S. Manichanh C. (2010). A human gut microbial gene catalogue established by metagenomic sequencing. Nature 464, 59–65 10.1038/nature08821 20203603 PMC3779803

[B94] Quan Z. Sun P. Lin G. Xi R. (2013). TSC1/2 regulates intestinal stem cell maintenance and lineage differentiation through Rheb-TORC1-S6K but independently of nutritional status or Notch regulation. J. Cell Sci. 126, 3884–3892 10.1242/jcs.125294 23843608

[B95] Ren C. Webster P. Finkel S. E. Tower J. (2007). Increased internal and external bacterial load during Drosophila aging without life-span trade-off. Cell Metab. 6, 144–152 10.1016/j.cmet.2007.06.006 17681150

[B96] Ren F. Wang B. Yue T. Yun E.-Y. Ip Y. T. Jiang J. (2010). Hippo signaling regulates Drosophila intestine stem cell proliferation through multiple pathways. Proc. Natl. Acad. Sci. U.S.A. 107, 21064–21069 10.1073/pnas.1012759107 21078993 PMC3000252

[B97] Rera M. Bahadorani S. Cho J. Koehler C. L. Ulgherait M. Hur J. H. (2011). Modulation of longevity and tissue homeostasis by the Drosophila PGC-1 homolog. Cell Metab. 14, 623–634 10.1016/j.cmet.2011.09.013 22055505 PMC3238792

[B98] Rera M. Clark R. I. Walker D. W. (2012). Intestinal barrier dysfunction links metabolic and inflammatory markers of aging to death in Drosophila. Proc. Natl. Acad. Sci. U.S.A. 109, 21528–21533 10.1073/pnas.1215849110 23236133 PMC3535647

[B99] Roberts S. B. Rosenberg I. (2006). Nutrition and aging: changes in the regulation of energy metabolism with aging. Physiol. Rev. 86, 651–667 10.1152/physrev.00019.2005 16601270

[B100] Ryu J. H. Ha E. M. Lee W. J. (2010). Innate immunity and gut-microbe mutualism in Drosophila. Dev. Comp. Immunol. 34, 369–376 10.1016/j.dci.2009.11.010 19958789

[B101] Ryu J.-H. Ha E.-M. Oh C.-T. Seol J.-H. Brey P. T. Jin I. (2006). An essential complementary role of NF-kappaB pathway to microbicidal oxidants in Drosophila gut immunity. EMBO J. 25, 3693–3701 10.1038/sj.emboj.7601233 16858400 PMC1538556

[B102] Ryu J. H. Kim S. H. Lee H. Y. Bai J. Y. Nam Y. D. Bae J. W. (2008). Innate immune homeostasis by the homeobox gene caudal and commensal-gut mutualism in Drosophila. Science 319, 777–782 10.1126/science.1149357 18218863

[B103] Sackton T. B. Lazzaro B. P. Schlenke T. A. Evans J. D. Hultmark D. Clark A. G. (2007). Dynamic evolution of the innate immune system in Drosophila. Nat. Genet. 39, 1461–1468 10.1038/ng.2007.60 17987029

[B104] Schloissnig S. Arumugam M. Sunagawa S. Mitreva M. Tap J. Zhu A. (2013). Genomic variation landscape of the human gut microbiome. Nature 493, 45–50 10.1038/nature11711 23222524 PMC3536929

[B105] Shaw R. L. Kohlmaier A. Polesello C. Veelken C. Edgar B. A. Tapon N. (2010). The Hippo pathway regulates intestinal stem cell proliferation during Drosophila adult midgut regeneration. Development 137, 4147–4158 10.1242/dev.052506 21068063 PMC2990206

[B106] Shin S. C. Kim S.-H. You H. Kim B. Kim A. C. Lee K.-A. (2011). Drosophila microbiome modulates host developmental and metabolic homeostasis via insulin signaling. Science 334, 670–674 10.1126/science.1212782 22053049

[B107] Sieber M. H. Thummel C. S. (2012). Coordination of triacylglycerol and cholesterol homeostasis by DHR96 and the Drosophila LipA homolog magro. Cell Metab. 15, 122–127 10.1016/j.cmet.2011.11.011 22197324 PMC3253980

[B108] Silverman N. Zhou R. Erlich R. L. Hunter M. Bernstein E. Schneider D. (2003). Immune activation of NF-kappaB and JNK requires Drosophila TAK1. J. Biol. Chem. 278, 48928–48934 10.1074/jbc.M304802200 14519762

[B109] Staley B. K. Irvine K. D. (2010). Warts and Yorkie mediate intestinal regeneration by influencing stem cell proliferation. Curr. Biol. 20, 1580–1587 10.1016/j.cub.2010.07.041 20727758 PMC2955330

[B110] Stecher B. Maier L. Hardt W.-D. (2013). ‘Blooming’ in the gut: how dysbiosis might contribute to pathogen evolution. Nat. Rev. Microbiol. 11, 277–284 10.1038/nrmicro2989 23474681

[B111] Storelli G. Defaye A. Erkosar B. Hols P. Royet J. Leulier F. (2011). Lactobacillus plantarum Promotes Drosophila Systemic Growth by Modulating Hormonal Signals through TOR-Dependent Nutrient Sensing. Cell Metab. 14, 403–414 10.1016/j.cmet.2011.07.012 21907145

[B112] Takashima S. Mkrtchyan M. Younossi-Hartenstein A. Merriam J. R. Hartenstein V. (2008). The behaviour of Drosophila adult hindgut stem cells is controlled by Wnt and Hh signalling. Nature 454, 651–655 10.1038/nature07156 18633350

[B113] Thevenon D. Engel E. Avet-Rochex A. Gottar M. Bergeret E. Tricoire H. (2009). The Drosophila ubiquitin-specific protease dUSP36/Scny targets IMD to prevent constitutive immune signaling. Cell Host Microbe 6, 309–320 10.1016/j.chom.2009.09.007 19837371

[B114] Tzou P. Ohresser S. Ferrandon D. Capovilla M. Reichhart J. M. Lemaitre B. (2000). Tissue-specific inducible expression of antimicrobial peptide genes in Drosophila surface epithelia. Immunity 13, 737–748 10.1016/S1074-7613(00)00072-8 11114385

[B115] Uronis J. M. Muhlbauer M. Herfarth H. H. Rubinas T. C. Jones G. S. Jobin C. (2009). Modulation of the intestinal microbiota alters colitis-associated colorectal cancer susceptibility. PLoS ONE 4:e6026 10.1371/journal.pone.0006026 19551144 PMC2696084

[B116] Wong C. N. Ng P. Douglas A. E. (2011). Low-diversity bacterial community in the gut of the fruitfly *Drosophila melanogaster*. Environ. Microbiol. 13, 1889–1900 10.1111/j.1462-2920.2011.02511.x 21631690 PMC3495270

[B117] Wullaert A. Bonnet M. C. Pasparakis M. (2011). NF-κ B in the regulation of epithelial homeostasis and inflammation. Cell Res. 21, 146–158 10.1038/cr.2010.175 21151201 PMC3193399

[B118] Xavier R. J. Podolsky D. K. (2007). Unravelling the pathogenesis of inflammatory bowel disease. Nature 448, 427–434 10.1038/nature06005 17653185

[B119] Xu N. Wang S. Q. Tan D. Gao Y. Lin G. Xi R. (2011). EGFR, Wingless and JAK/STAT signaling cooperatively maintain Drosophila intestinal stem cells. Dev. Biol. 354, 31–43 10.1016/j.ydbio.2011.03.018 21440535

[B120] Zaidman-Remy A. Herve M. Poidevin M. Pili-Floury S. Kim M. S. Blanot D. (2006). The Drosophila amidase PGRP-LB modulates the immune response to bacterial infection. Immunity 24, 463–473 10.1016/j.immuni.2006.02.012 16618604

[B121] Zhou F. Rasmussen A. Lee S. Agaisse H. (2013). The UPD3 cytokine couples environmental challenge and intestinal stem cell division through modulation of JAK/STAT signaling in the stem cell microenvironment. Dev. Biol. 373, 383–393 10.1016/j.ydbio.2012.10.023 23110761 PMC3534909

